# Magnetic Resonance–Guided Focused Ultrasound Treatment for Essential Tremor: A Single‐Center Experience

**DOI:** 10.1002/mdc3.70012

**Published:** 2025-02-19

**Authors:** Victor S. Hvingelby, Pernille Kjeldsen, Bo Bergholt, Gaston Andres Schechtmann, Erik Hvid Danielsen, Mette Møller, Erik Lisbjerg Johnsen, Skirmante Mardosiene, Torben Ellegaard Lund, Dora Grauballe, Michael Geneser, Tina Vincens Sørensen, Lisa Østergaard Bak, Martin Andreasen, Anne Sofie Møller Andersen, Lone Andersen, Kaare Meier, Niels Juhl, Alp Tankisi, Bo Jespersen, Christian Fenger Eriksen, Mads Rasmussen, Winnie Bechmann Eriksen, Birgitte Barrutia, Mette Kromann, Ida Baandrup, Jette Bjørn, Rie Stjernholm, Charlotte Bræmer‐Madsen, Signe Mygdal Jørgensen, Jens Christian Hedemann Sørensen, Andreas Nørgaard Glud

**Affiliations:** ^1^ Department of Neurosurgery Aarhus University Hospital Aarhus Denmark; ^2^ Center of Experimental Neuroscience, Aarhus University Hospital Aarhus Denmark; ^3^ Department of Health Clinical Institute, Aarhus University Aarhus Denmark; ^4^ Department of Neurology Aarhus University Hospital Aarhus Denmark; ^5^ Department of Neurology Rigshospitalet Bispebjerg Denmark; ^6^ CFIN, Aarhus University Hospital Aarhus Denmark; ^7^ Department of Radiology Section Neuro, Aarhus University Hospital Aarhus Denmark; ^8^ Danish Neuroscience Center Aarhus University Hospital Aarhus Denmark; ^9^ Department of Anaesthesiology Aarhus University Hospital Aarhus Denmark

**Keywords:** essential tremor, focused ultrasound, magnetic resonance imaging

## Abstract

**Background:**

Essential tremor is the most common hyperkinetic movement disorder. Magnetic resonance imaging–guided focused ultrasound (MRgFUS) has emerged as second‐line therapy.

**Objective:**

The aim was to obtain the results of the first 108 patients treated with MRgFUS in Denmark.

**Methods:**

Data were entered in a quality assurance database at baseline and 3, 6, and 12 months. Clinician‐ and patient‐rated treatment efficacy was evaluated using the Fahn–Marin–Tolosa (FMT) scale and the Patient Global Impression of Change.

**Results:**

A total of 108 persons have currently been treated. Tremor improved by a total mean 6.39 points (95% confidence interval [CI]: 5.01;7.76, *P* < 0.00001) and 9.63 points (95% CI: 7.60;11.66, *P* < 0.00001), 10.42 (95% CI: 9.06;11.79, *P* < 0.00001), and 26.45 (95% CI: 22.46;30.43, *P* < 0.00001) for FMT parts A, B, and C, respectively, at 3 months. Side effects occurred in 65.7% of patients at 3 months and 33.7% at 12 months.

**Conclusion:**

Our findings are in line with existing evidence. Questions regarding persistence of gait‐ and balance‐related side effects remain.

Essential tremor (ET) is the most common hyperkinetic movement disorder, defined according to the Movement Disorder Society (MDS) Consensus Statement on Tremor.^1^, ^2^ Although not considered to affect life expectancy, ET has significant impact on quality of life and activities of daily living.^3^ In recent years, magnetic resonance imaging–guided focused ultrasound (MRgFUS) has emerged as an advanced therapy for medically refractory ET.^4^

Long‐term outcomes and efficacy of MRgFUS have been documented.^5^, ^6^, ^7^ Here, we add to the knowledge base of MRgFUS in ET by reporting a cohort of Danish and Faroese patients having received MRgFUS for tremor.

## Patients and Methods

Results are based on data entered into a quality assurance database. The first patient was entered in June 2022. The final patient in this report was treated in February 2024. Treatments are ongoing, and additional data are being collected on a rolling basis, and patients, including those reported here, are followed up.

### Inclusion Criteria

- ET diagnosis according to MDS consensus criteria, diagnosed by a movement disorder specialist neurologist
- Unsatisfactory pharmacological response defined as either
  No or incomplete response to at least 3 medications
  Pharmacological management contraindicated or causing unacceptable side effects

Exclusion criteria are similar to general criteria for undergoing surgery or stereotaxic procedures and MRI such as metal implants, bleeding disorders, Parkinsons or Parkinson Plus syndromes, structural brain abnormalities affecting ablation, or a skull density ratio <0.35.

Patients are referred for multidisciplinary evaluation for specialized treatment of ET. The decision to offer MRgFUS is performed at the discretion of the multidisciplinary team.

Treatment is performed according to standardized procedure^8^ (Supporting Information).

Prior to MRgFUS, patients are assessed by a movement disorder neurologist. Baseline and demographic data are collected in the database, including occupational status, medications, smoking, alcohol intake, and related measures. This is repeated at 6 and 12 months only.

The primary clinician‐based outcome of tremor severity is the Fahn–Marin–Tolosa (FMT) scale at baseline and at 3 and 12 months.^9^ The primary outcome of patient‐assessed efficacy is the Patient Global Impression of Change (PGIC) and secondarily the Quality of Life in Essential Tremor (QUEST) questionnaire at baseline and at 3, 6, and 12 months.^10^ General QoL is assessed using the Short Form 36 (SF‐36) at baseline and at 3, 6, and 12 months.^11^ At baseline patients state what they perceive to be the main symptom of tremor and a treatment goal, that is, an ability they wish to regain or activity they would like to pursue. At 3, 6, and 12 months, they evaluate whether their treatment goal is met. No constraints are in place regarding the statement of this goal. At each follow‐up patients are asked whether they would recommend treatment to others.

Treatment tolerability is assessed primarily as the presence of side effects after treatment and secondarily as periprocedural side effects. Here, “unsteady gait” denotes any report by patients or clinicians, and “ataxia” denotes a specific observation of ataxia by a clinician.

For numerical rating scales, including the FMT, QUEST, SF‐36, and subsections, outcome is evaluated as the mean of individual changes from baseline to follow‐up. Mean percentage, or relative, changes are calculated as the mean of individual change in percentage. The same procedure is performed for medication dosages.

For the PGIC, the fraction of the cohort indicating each response option at each time point is calculated. Treatment effect at 3 months is evaluated using paired *t*‐tests and across all time‐points using a repeated‐measures analysis of variance (ANOVA), with baseline score entered as covariate. For overall QoL, end‐of‐treatment scores are compared against baseline using unpaired *t*‐tests. Regarding patients' treatment goals, the fraction of the cohort indicating that this had been attained is calculated for each time point. Health‐behavior indicators are percentage of participants engaging in volunteer work and self‐reported units of alcohol per week, and, for people who self‐identified as smokers, the number of cigarettes per day, self‐reported minutes of exercise per week, and number of occasions during a standard week in which patients engaged in physical activity evaluated on a 5‐point Likert scale. These outcomes are reported at baseline and at 3 and 12 months.

## Results

A total of 108 patients had been treated at the time of writing and are included as the baseline cohort. At the time of writing, 90 patients had reached the 3‐month follow‐up, 70 patients the 6‐month follow‐up, and 28 patients the 12‐month follow‐up.

Demographic and treatment variables are presented in Tables S1 and S2 and Figure S1. There was an overweight of male compared to female patients (80/108). The most frequent comorbidity was cardiac disease (44/108) followed by type 2 diabetes (17/108). A total of 29.6% experienced minor gait problems and 20.4% marked gait or balance issues at baseline assessment. Baseline assessor‐rated tremor severity, according to the FMT, was 49 ± 13 points, with large intersubject variation in terms of tremor distribution and severity (Table S1). Baseline ratings of tremor severity assessed according to the different subdomains of the QUEST showed that the most severely affected subdomain was physical health, with a mean score of 75.5 prior to surgery.

**FIG. 1:**
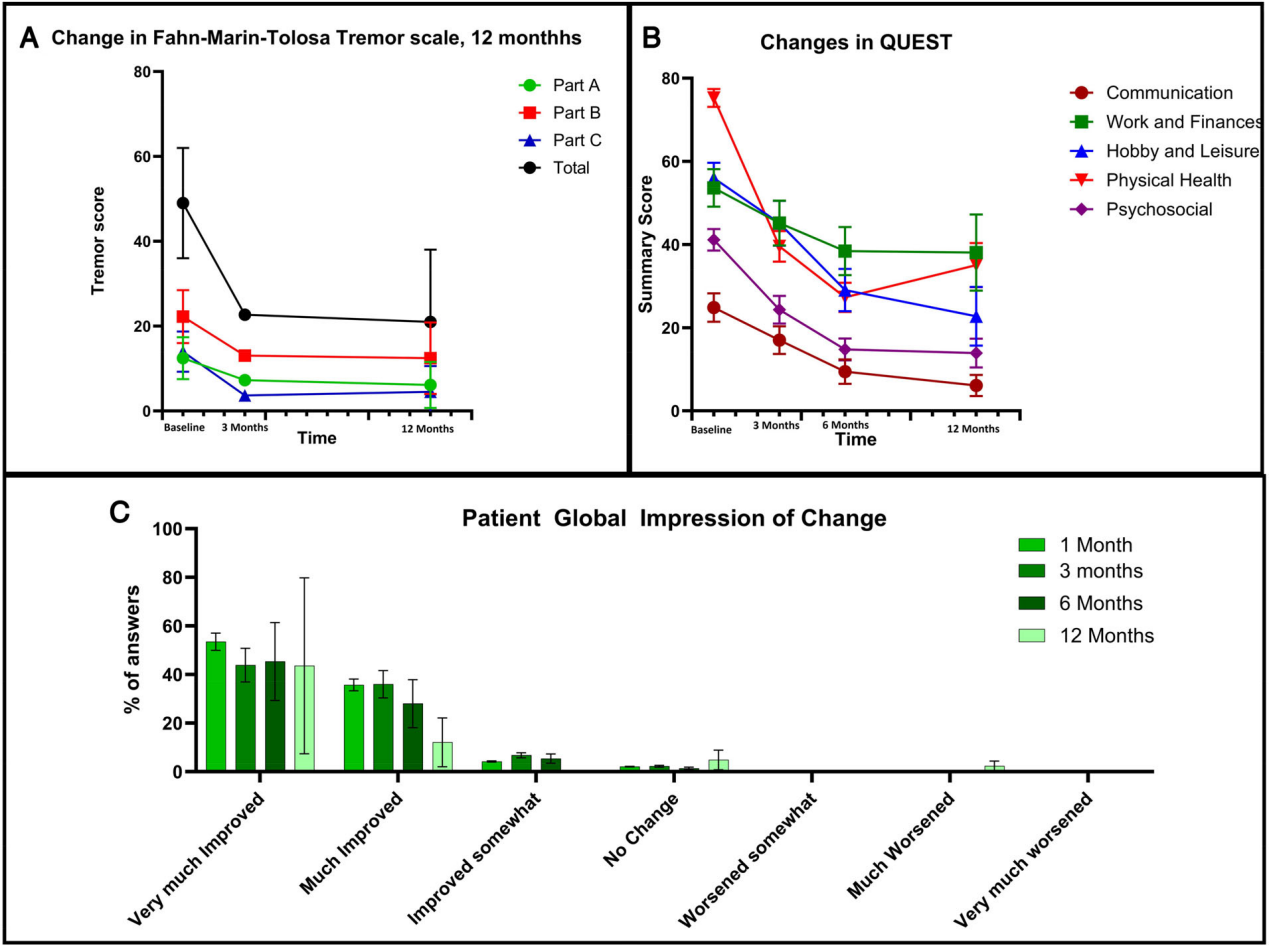
Changes in assessor‐ and patient‐rated outcomes across the first year after treatment. (**A**) The changes between baseline, 3 months, and 12 months according to the Fahn–Marin–Tolosa (FMT) scale. (**B**) The changes between baseline, 3 months, 6 months, and 12 months according to the patient‐rated Quality of Life in Essential Tremor (QUEST) scale and (**C**) the Patient Global Impression of Change (PGIC) at 1, 3, 6, and 12 months.

With regard to assessor‐based changes in tremor, treatment has statistically and clinically significant positive effects. The mean change on the FMT between baseline and 3 months was 6.39 point improvement (95% confidence interval [CI]: 5.01;7.76, *P* < 0.00001), 9.63 point improvement (95% CI: 7.60;11.66, *P* < 0.00001), 10.42 point improvement (95% CI: 9.06;11.79, *P* < 0.00001), and 26.45 point improvement (95% CI: 22.46;30.43, *P* < 0.00001) for parts A, B, and C and total score, respectively. An RM‐ANOVA performed across all time points indicated that effects were sustained after 12 months (Fig. 1; Table S3).

For patient‐based ratings, most experienced a positive effect of treatment according to the PGIC, with 48.8% indicating very much improved and 40.0% much improved at 3 months. Only 7.5% indicated to be improved somewhat and 2.5% no change (Fig. 1). No patients indicated deterioration. These results are also reflected in both the results of the QUEST and SF‐36 (Fig. 1; Table S3).

Additionally, 87.1% of patients stated that the treatment goal they had set for themselves prior to treatment is reached. Overwhelmingly, patients indicated the main symptom to be very much improved (48.8%), much improved (39.3%), or slightly improved (8.3%), and 95.8% indicated that they would recommend the treatment to others after 3 months.

**FIG. 2:**
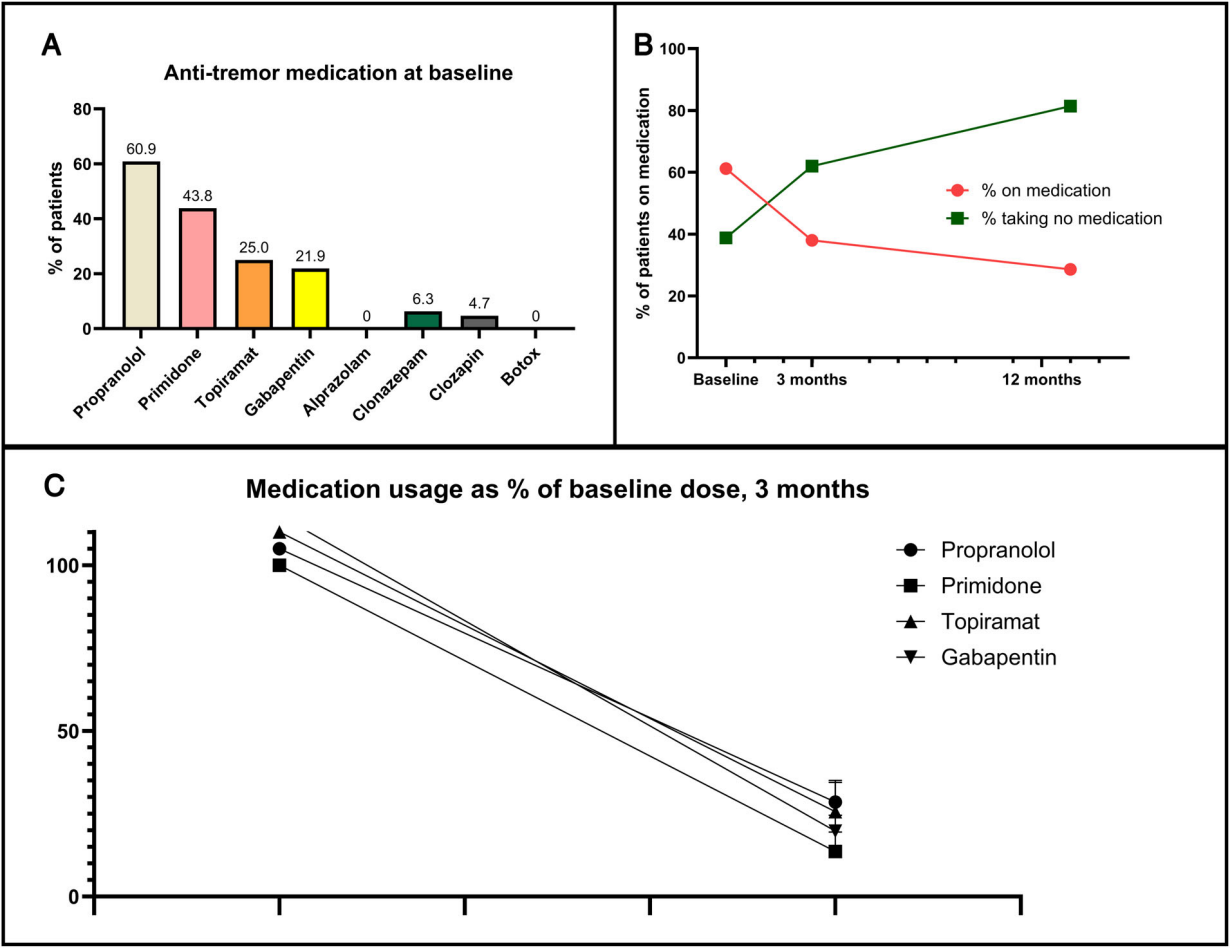
Changes in the use of medication after treatment in the first 12 months after treatment. (**A**) The frequency of ingestion of common antitremor medications. (**B**) The percentage of patients taking and not taking antitremor medications, respectively, at baseline, 3 months, and 12 months. (**C**) The mean of individual medication usage as a percentage of use at baseline after 3 months where dose at baseline is set at 100%.

The use of medication for tremor decreased for all types of medication for which sufficient data was available after 6 months (Fig. 2).

Slight differences in socioeconomic factors were apparent after 3 months. However, these changes were not statistically significant in this cohort (Fig. S2).

Procedure‐related side effects were mostly mild, transient, and self‐limited.

In total, 82.2% of patients experienced side effects related to the procedure. Most commonly brief disequilibrium was encountered in 59.8% (56/93) of patients. A total of 15.1% experienced paresthesia and 1.1% transient dysmetria. No patients experienced paresis or weakness in any body part. Further, 2.2% experienced dysarthria. Other side effects included nausea (9.7%), warm sensation of head (7.5%), headache (4.3%), back pain (1.1%), and jerks of a limb (2.2%).

Persistent side effects of treatment are also reported. At 3 months 65.7% experienced some side effect, most commonly unsteady gait, as reported by 44.3% of patients (20.4%–29.6% at baseline). At 12 months 33.3% reported this symptom. Paresthesia was reported to persist by 13.9% of patients (13.9% face, lips, and tongue; 7.6% hands and fingers; and 1.3% legs). This was reported at 11.4% at 12 months (10.0% face, lips, and tongue; 7.1% hands and fingers; and 2.9% legs). Dysgeusia was reported to persist in 21.8% of patients at 3 months and 8.6% at 12 months. Ataxia was noted in 5.3% of patients at 3 months and 8.8% at 12 months. Overall, at 12 months 33.7% indicated at least 1 side effect, meaning 59.0% of side effects reported at 3 months had subsided.

## Discussion

In this report, we present treatment outcomes from a cohort of Danish and Faroese ET patients treated with MRgFUS in Denmark. Patients exhibited good effects of the treatment and marked symptom improvement after 3 months. Patients had less tremor as measured using the FMT scale. Patients also had subjectively less tremor and challenges related to tremor as assessed using the PGIC and QUEST and better QoL as assessed using the SF‐36. These effects were sustained after 12 months (Table S3); however, data for this time point are currently limited, as data collection is still ongoing.

Patients generally have favorable views of treatment, and 87.1% of patients stated their treatment goals were met. A number of patients were able to engage in more health‐promoting activities after treatment, including physical exercise, social functions, and work‐related tasks. Additionally, more were able to engage in activities such as volunteer work (25.9% at 3 months vs. 8.4% at baseline).

The side effects of treatment were generally manageable. However, a considerable number of patients reported gait problems subsequent to treatment. Importantly, however, as evident from the baseline assessment, a similar proportion experienced some form of gait or balance problem at baseline, based on neurological evaluation with straight line test and pull back test. The apparent increase in prevalence of ataxia between 3 and 12 months is not expected to be related to thalamotomy. The exact reason for this apparent increase in our cohort is thus at most speculative. It may be due to progression of baseline instability related to factors other than tremor or to factors not targeted by thalamotomy. Alternatively, it may be an artifact as the 3‐ and 12‐month cohorts differ. In this regard, the lack of stratification of severity of these side effects is a limitation of the present study. It does however point to the need for more attention being afforded instability and ataxia in ET and after MRgFUS in the future. Analysis of correlations between lesion size and location with characteristics of side effects was not possible as this was not part of the protocolized data collection but represents an important avenue of potential future research. We plan to follow our cohort for 5 and 10 years to describe the longitudinal treatment outcome and to hopefully improve counseling, treatment, and general patient care in the future.

Our findings are consistent with similar results previously published. Questions regarding management and persistence of gait‐ and balance‐related side effects remain. This represents a question for future research and a potential opportunity for enhanced rehabilitation.

## Author Roles

(1) Research project: A. Conception, B. Organization, C. Execution; (2) Statistical analysis: A. Design, B. Execution, C. Review and critique; (3) Manuscript preparation: A. Writing of the first draft, B. Review and critique.

V.S.H.: 1A, 1B, 1C, 2A, 2B, 2C, 3A, 3B

P.K.: 1C, 2C, 3B

Bo Bergholt: 1A, 1B, 1C, 2C, 3B

G.A.S.: 1A, 1B, 1C, 2C, 3B

E.H.D.: 1A, 1B, 1C, 2C, 3B

M.M.: 1A, 1B, 1C, 2C, 3B

E.L.J.: 1A, 1B, 1C, 2C, 3B

S.M.: 1C, 2C, 3B

T.E.L.: 1C, 2C, 3B

D.G.: 1C, 2C, 3B

M.G.: 1C, 2C, 3B

T.V.S.: 1C, 2C, 3B

L.Ø.B.: 1C, 2C, 3B

M.A.: 1C, 2C, 3B

A.S.M.A.: 1C, 2C, 3B

L.A.: 1C, 2C, 3B

K.M.: 1B, 1C, 2C, 3B

N.J.: 1C, 2C, 3B

A.T.: 1C, 2C, 3B

B.J.: 1A, 1B, 1C, 2C, 3B

C.F.E.: 1C, 2C, 3B

M.R.: 1C, 2C, 3B

W.B.E.: 1C, 2C, 3B

Birgitte Barrutia: 1C, 2C, 3B

M.K.: 1C, 2C, 3B

I.B.: 1C, 2C, 3B

J.B.: 1C, 2C, 3B

R.S.: 1C, 2C, 3B

C.B.‐M.: 1C, 2C, 3B

S.M.J.: 1C, 2C, 3B

J.C.H.S.: 1A, 1B, 1C, 2C, 3B

A.N.G.: 1A, 1B, 1C, 2A, 2B, 2C, 3A, 3B

## Disclosures

**Ethical Compliance Statement:** The present project was initiated as an extension of a quality assurance database. As such, the project was conducted without the need for prior institutional review board approval. All patients treated, and subsequently entered into the database, provided informed consent, which is documented in the electronic patient record. We confirm that we have read the journal's position on issues involved in ethical publication and affirm that this work is consistent with those guidelines.

**Funding Sources and Conflicts of Interest:** The authors declare that there are no conflicts of interest relevant to this work. No specific funding was received for this work.

**Financial Disclosures for the Previous 12 Months:** The authors declare that there are no additional disclosures to report.

## Supporting information


**Supplementary Materials**: A description of the steps of the procedure, including preparation, imaging, and target determination.

**Table S1.** Demographic and tremor characteristics at baseline. MoCA, Montreal Cognitive Assessment; QUEST, Quality of Life in Essential Tremor Rating Scale; SF‐36, Short Form 36.

**Table S2.** Data describing the details of the procedure. For each outcome, the result is given as the mean ± standard deviation. Temperatures are provided as degree Celsius.

**Table S3.** Change in tremor characteristics and indices of quality of life and health‐related activities at 3 months, calculated as the mean change in scores/percentages and across all time points, calculated as a repeated‐measures analysis of variance (RM‐ANOVA). QUEST, Quality of Life in Essential Tremor Rating Scale; SF‐36, Short Form 36.

**Figure S1.** Distribution and severity of tremor in the 4 extremities and head, face, and/or voice at baseline, according to the Fahn–Marin–Tolosa (FMT) scale, stratified by subsection.

**Figure S2.** Changes in work and exercise habits between baseline and 3 months. (**A**) Changes in frequency of exercise, with black bars indicating baseline and gray bars indicating 3 months. (**B**) A violin plot of changes in minutes of exercise per week. (**C**) A pie chart of the portion of the cohort that engages in volunteer work at baseline and 3 months, respectively.

## Data Availability

The data that support the findings of this study are available on request from the corresponding author. The data are not publicly available due to privacy or ethical restrictions.
