# Supplementary material for: Magnetic Resonance–Guided Focused Ultrasound Treatment for Essential Tremor: A Single‐Center Experience
Source: Mov Disord Clin Pract. 2025 Feb 19;12(7):922–7. doi: 10.1002/mdc3.70012 (PMC12274985; doi:10.1002/mdc3.70012)
Supplement: Supplementary file 1 — Supplementary Materials: A description of the steps of the procedure, including preparation, imaging, and target determination. [file MDC3-12-922-s004.docx]

Description of the procedure:

The treatment was performed according to the following standardized procedure:

The patient was prepared under cover of local anesthesia applied to the thoroughly shaved scalp. An InsightTech-stereotaxic frame was fixated to the skull with (4) integra skull pins prior to the patient being placed on the bed for the procedure.

A preprocedural T1 weighted MRI sequence was obtained for final procedural planning. Based on the pre-procedural MRI, the AC-PC line was ascertained, and target traced. The distance to the AC-PC line was calculated as well as the distance to the boarder of the third ventricle. A minimal capsular distance of 3.0 mm was applied.

Prior to procedure commencement, MRI and ultrasound equipment was calibrated, and, once the patient was placed within the scanner, a baseline MR scan was performed and fused with prior imaging, precision of sonication hardware was tested by sonications with test heating at low temperatures in three directions, with adjustments performed as needed. Subsequently, a higher energy verification sonication was applied, allowing a transitory effect on tremor, and possibility to evaluate side effects.

A target standard was chosen 1 mm behind the AC-PC line. DTI imaging was not routinely performed in patients, and instead treatment and target optimization was done based on running assessment of clinical efficacy.

Following verification sonication, energy was increased to levels expected to achieve clinical efficacy and permanence (consolidation). The energy levels for this were such that permanent lesioning of the VIM nucleus was expected, with either 50C in 20 seconds, 2 ablations with minimum 55C or one above 58C. After each therapeutic sonication, the patient was assessed for side-effects, tolerability and clinical efficacy by the clinical team, including a specialist neurologist, a neurosurgeon, and a neuroanesthesiologist. The total number of sonications (alignment+verification+therapeutic) were recorded.
