# Supplementary material for: Magnetic Resonance–Guided Focused Ultrasound Treatment for Essential Tremor: A Single‐Center Experience
Source: Mov Disord Clin Pract. 2025 Feb 19;12(7):922–7. doi: 10.1002/mdc3.70012 (PMC12274985; doi:10.1002/mdc3.70012)
Supplement: Supplementary file 2 — Table S1. Demographic and tremor characteristics at baseline. MoCA, Montreal Cognitive Assessment; QUEST, Quality of Life in Essential Tremor Rating Scale; SF‐36, Short Form 36. [file MDC3-12-922-s001.docx]

| Supplementary Table 1: Demographic and baseline clinical characteristics of cohort | | | |
| --- | --- | --- | --- |
| n | | 108 | |
| Gender (F/M) | | (28 / 80) | |
| Age (years) | | 72.44 ± 8.59 | |
| MoCA | | 26.16 ±2.78 | |
| Comorbidity (n, %) | |  | |
| Type 1 Diabetes | | 6 (11.3%) | |
| Type 2 Diabetes | | 17 (32.1%) | |
| Diabetic Polyneuropathy | | 5 (9.4%) | |
| Polyneuropathy, not otherwise specified | | 3 (5.7%) | |
| Cardiac disease | | 44 (83%) | |
| Treated with Beta-blocker? | | 14 (34.1%) | |
| Gait/Balance Problems | |  | |
| Normal/No Balance problems | | 49 (42.59%) | |
| Near Normal / Minor problems | | 32 (29.63%) | |
| Gait Problems / Poor gait | | 22 (20.37%) | |
| No information | | 5 (7.4%) | |
| Medication (n (%)) | Mean dose, SD |  | |
| Propranolol | | 39 (60.9 %) | 118.72 mg ±66.62 mg |
| Primidone | | 28 (43.8 %) | 150 mg ±129.64 mg |
| Topiramat | | 16 (25 %) | 80 mg ±70.38 |
| Gabapentin | | 14 (21.9 %) | 1264.3 mg ±1047.1mg |
| Alprazolam | | 0 |  |
| Clonazepam | | 4 (6.3 %) | N/A |
| Clozapine | | 3 (4.7 %) | 150 mg ±132.29mg |
| Botox inj. | | 0 |  |
| Fahn-Marin-Tolosa | |  | |
| Part A | | 12.43 ±4.94 | |
| Part B | | 22.25 ±6.25 | |
| Part C | | 14.00 ±4.75 | |
| Total | | 49.00 ±13.00 | |
| QUEST | |  | |
| Overall health | | 66.11 ±22.15 | |
| Overall QoL | | 58.47 ±25.23 | |
| Tremor hours / day | | 15.79 h ±4.85 h | |
| Communication | | 8.33 IQR: 33.33, range 0-100 | |
| Work and Finance | | 42.71 IQR: 75, range 0-100 | |
| Hobbies and Leisure | | 36.09 ±5.23 | |
| Physical health | | 75.5 ±21.89 | |
| Psychosocial | | 41.06 ±35.95 | |
| SF-36 | |  | |
| Physical Functioning | | 68.11 ±25.65 | |
| Limitations due to Physical Health | | 48.15 ±41.04 | |
| Limitations due to emotional problems | | 55.83 ±43.98 | |
| Energy/Fatigue | | 45.43 ±24.46 | |
| Emotional Well-Being | | 30.93 ±21.07 | |
| Social Functioning | | 75.47 ±26.57 | |
| Pain | | 74.51 ±25.27 | |
| General Health | | 38.83 ±18.05 | |
| Work | |  | |
| Unemployed | | 1 (0.9%) | |
| Retired | | 83 (76.9%) | |
| Early retirement due to disability | | 10 (9.3%) | |
| Senior job | | 2 (1.9%) | |
| Part time work | | 1 (0.9%) | |
| Full time work | | 11 (10.2%) | |
| Volunteer work | | 11 (10.2%) | |
| Alcohol per week | | 4 (IQR: 10) | |
| Tobacco | | 27 (25.2%) | |
| Cigarettes per day, smokers | | 14.22 ±6.82 | |
| Exercise | |  | |
| Several times per day | | 7 (6.5%) | |
| Once per day | | 31 (28.7)% | |
| 3-5 times per week | | 25 (23.1%) | |
| Once per week | | 11 (10.2%) | |
| Less than once per week | | 34 (31.5%) | |
| Approximate minutes of exercise per week | | 120 min (IQR: 246.5 min) | |
