# Supplementary material for: Magnetic Resonance–Guided Focused Ultrasound Treatment for Essential Tremor: A Single‐Center Experience
Source: Mov Disord Clin Pract. 2025 Feb 19;12(7):922–7. doi: 10.1002/mdc3.70012 (PMC12274985; doi:10.1002/mdc3.70012)
Supplement: Supplementary file 3 — Table S2. Data describing the details of the procedure. For each outcome, the result is given as the mean ± standard deviation. Temperatures are provided as degree Celsius. [file MDC3-12-922-s005.docx]

| Supplementary Table 2: Procedure Related Data | | |
| --- | --- | --- |
| Distance from therapeutic sonication(s) to third ventricle | 10.95 mm ±0.65 mm |  |
| Distance from therapeutic sonication to midline | 14.48 mm ±1.62 mm |  |
| Skull Density Ratio (SDR) | 0.53 ±0.10 |  |
| Number of available sonication heads | 925.98 ±44.04 |  |
| Skull area | 365.14 mm^2^ ± 31.14mm^2^ |  |
| Number of therapeutic sonications | 2.35 ± 1.89 |  |
| Mean temperature of therapeutic sonications | 54.86 °C ±5.63 °C |  |
| Mean energy delivered per therapeutic sonication | 13600.59 J ± 9756.62 J |  |
| Mean duration of therapeutic sonications | 20.16 s ±13.08 s |  |
| Total time in scanner | 112.21 minutes ± 35.43 minutes |  |
