# Supplementary material for: Magnetic Resonance–Guided Focused Ultrasound Treatment for Essential Tremor: A Single‐Center Experience
Source: Mov Disord Clin Pract. 2025 Feb 19;12(7):922–7. doi: 10.1002/mdc3.70012 (PMC12274985; doi:10.1002/mdc3.70012)
Supplement: Supplementary file 4 — Table S3. Change in tremor characteristics and indices of quality of life and health‐related activities at 3 months, calculated as the mean change in scores/percentages and across all time points, calculated as a repeated‐measures analysis of variance (RM‐ANOVA). QUEST, Quality of Life in Essential Tremor Rating Scale; SF‐36, Short Form 36. [file MDC3-12-922-s003.docx]

| Supplementary Table 3: Clinical effects of MRgFUS | | | | | | | |
| --- | --- | --- | --- | --- | --- | --- | --- |
| Outcome | Mean difference at 3 months | Mean change in % | | significance at 3 months | | RM-ANOVA | |
| Medication* |  |  | |  | |  | |
| Propranolol | -98.54 (12.22) | -76.48% (6.55%) | | p<0.00001 | |  | |
| Primidone | -116.38 (20.13) | -86.43% (5.89%) | | p<0.00001 | |  | |
| Topiramat | -50.31 (10.82) | -84.38% (8.8%) | | p=0.0002 | |  | |
| Gabapentin | -1221.43 (287.31) | -95.24% (4.76%) | | p=0.0005 | |  | |
| Alprazolam | N/A | | | | | | |
| Clonazepam | N/A | | | | | | |
| Clozapine | N/A | | | | | | |
| Botox inj. | N/A | | | | | | |
| Fahn-Marin-Tolosa scale | See figure 1 | | | | | | |
| Part A | -6.75 (0.58) | 51.24 % (3.49%) | | p<0.00001 | | F(2,78)=66.35, p<0.00001 | |
| Part B | -9.21 (0.75) | 41.49 % (3.53%) | | p<0.00001 | | F(2,73)=83.14, p<0.00001 | |
| Part C | -10.35 (0.56) | 74.88% (3.42%) | | p<0.00001 | | F(2,77)=174.38, p<0.00001 | |
| Total | -26.32 (1.38) | 55.21% (2.57%) | | p<0.00001 | | F(2,73)=197.87, p<0.00001 | |
| QUEST |  |  | |  | |  | |
| Overall health | 5.67 (2.47) | 16.48% (5.72%) | | p=0.0125 | | F(4,221)=1.39,  p=0.2442 | |
| Overall QoL | 15.97 (3.09) | 44.76% (10.68%) | | p<0.0001 | | F(4,220)=12.91  p=0.0007 | |
| Tremor hours / day | -4.6 (1.05) | 19.40% (7.91%) | | p<0.0001 | | F(4,218)=13.25  p=0.0006 | |
| Subjective Tremor Severity | See Supplementary Figure 1 | | | | | | |
| Communication | -10.23 (4.85) | 29.08% (20.4%) | | p = 0.0194 | | F(4,222)=7.41  p=0.0086 | |
| Work and Finance | -1.54 (7.48) |  | | p=0.4185 | | F(4,221)=6.50  p=0.0136 | |
| Hobbies and Leisure | -5.56 (5.55) |  | | p=0.1601 | | F(4,222)=15.70  p=0.0002 | |
| Physical health | -36.08 (3.97) | 46.73% (5.24%) | | p<0.0001 | | F(4,222)=78.09  p<0.0001 | |
| Psychosocial | -16.50 (3.71) | 28.56& (13.49%) | | p<0.0001 | | F(4,219)=35.53  p<0.0001 | |
|  |  |  | |  | |  | |
| SF-36 |  |  | |  | |  | |
| Physical Functioning | 0.96 (2.12) | 13.40% (11.57%) | | p=0.675 | | F(3,135)=3.17  p=0.0266 | |
| Limitations due to Physical Health | 0.45 (5.12) | 4.16 % (11.34%) | | p=0.5351 | | F(3,136)=0.87  p=0.4558 | |
| Limitations due to emotional problems | 17.90 (6.09) | 10.78% (10.69%) | | p=0.0024 | | F(3,135)=5.18  p=0.002 | |
| Energy/Fatigue | 0.88 (2.54) | 14.78% (7.6%) | | p=0.5137 | | F(3,137)=1.24  p=0.2963 | |
| Emotional Well-Being | 6.36 (2.32) | 1.03 % (15.10%) | | p=0.0042 | | F(3,136)=7.36  p=0.0001 | |
| Social Functioning | 7.67 (3.23) | 22.34% (7.80%) | | p=0.0105 | | F(3,135)=4.05  p=0.0086 | |
| Pain | -3.20 (3.63) | 6.15% (8.17%) | | p=0.1911 | | F(3,139)=0.66  p=0.5804 | |
| General Health | 1.72 (2.70) | 18.00% (9.42%) | | p=0.2628 | | F(3,134)=0.10  p=0.95 | |
| Volunteer work | See Supplementary Figure 2 | | | | | | |
| Alcohol per week | -0.22 (0.53) | |  | | p=0.3378 | |  |
| Cigarettes per day, smokers | -0.125 (1.04) | | -4.7 % (9.0%) | | p = 0.4528 | |  |
| Approximate minutes of exercise per week | 14.76 (33.78) | |  | | p = 0.33 | |  |
