# Supplementary figures and images for: Magnetic Resonance–Guided Focused Ultrasound Treatment for Essential Tremor: A Single‐Center Experience
Source: Mov Disord Clin Pract. 2025 Feb 19;12(7):922–7. doi: 10.1002/mdc3.70012 (PMC12274985; doi:10.1002/mdc3.70012)

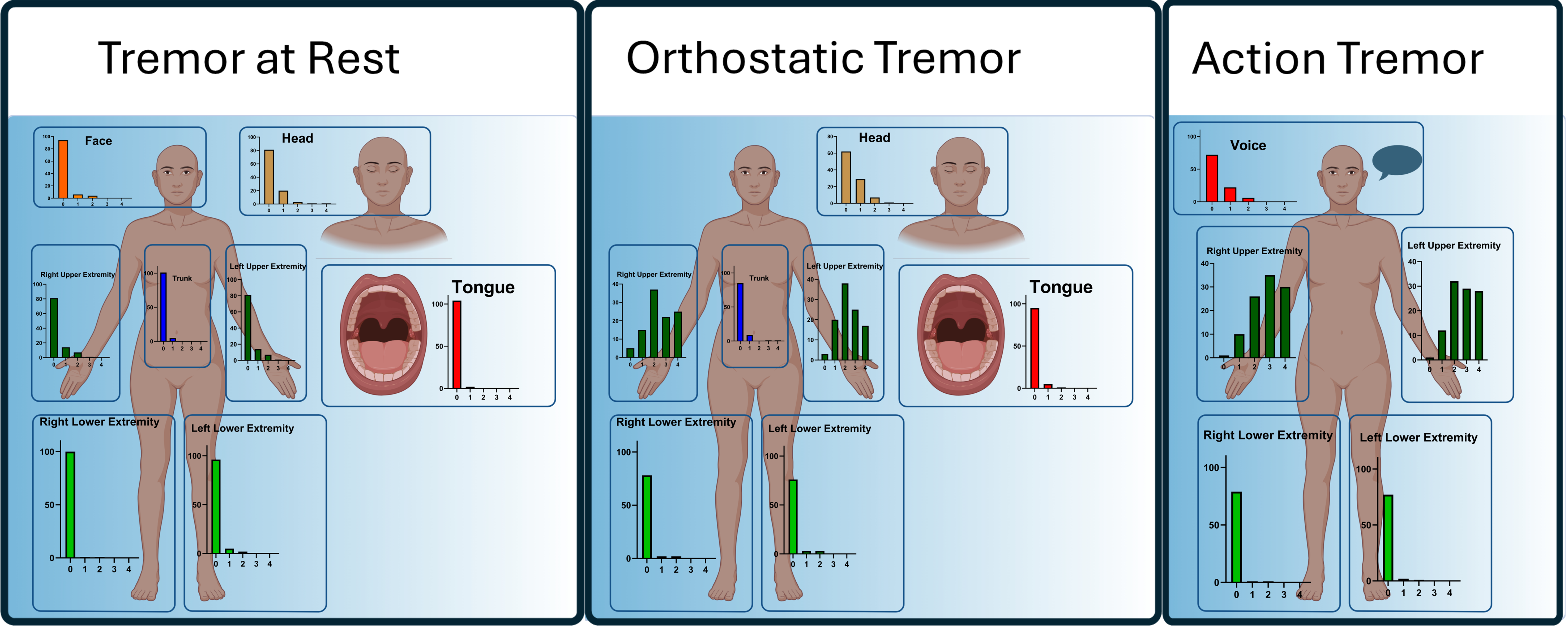

Supplement: Supplementary file 5 — Figure S1. Distribution and severity of tremor in the 4 extremities and head, face, and/or voice at baseline, according to the Fahn–Marin–Tolosa (FMT) scale, stratified by subsection. [file MDC3-12-922-s006.tif]

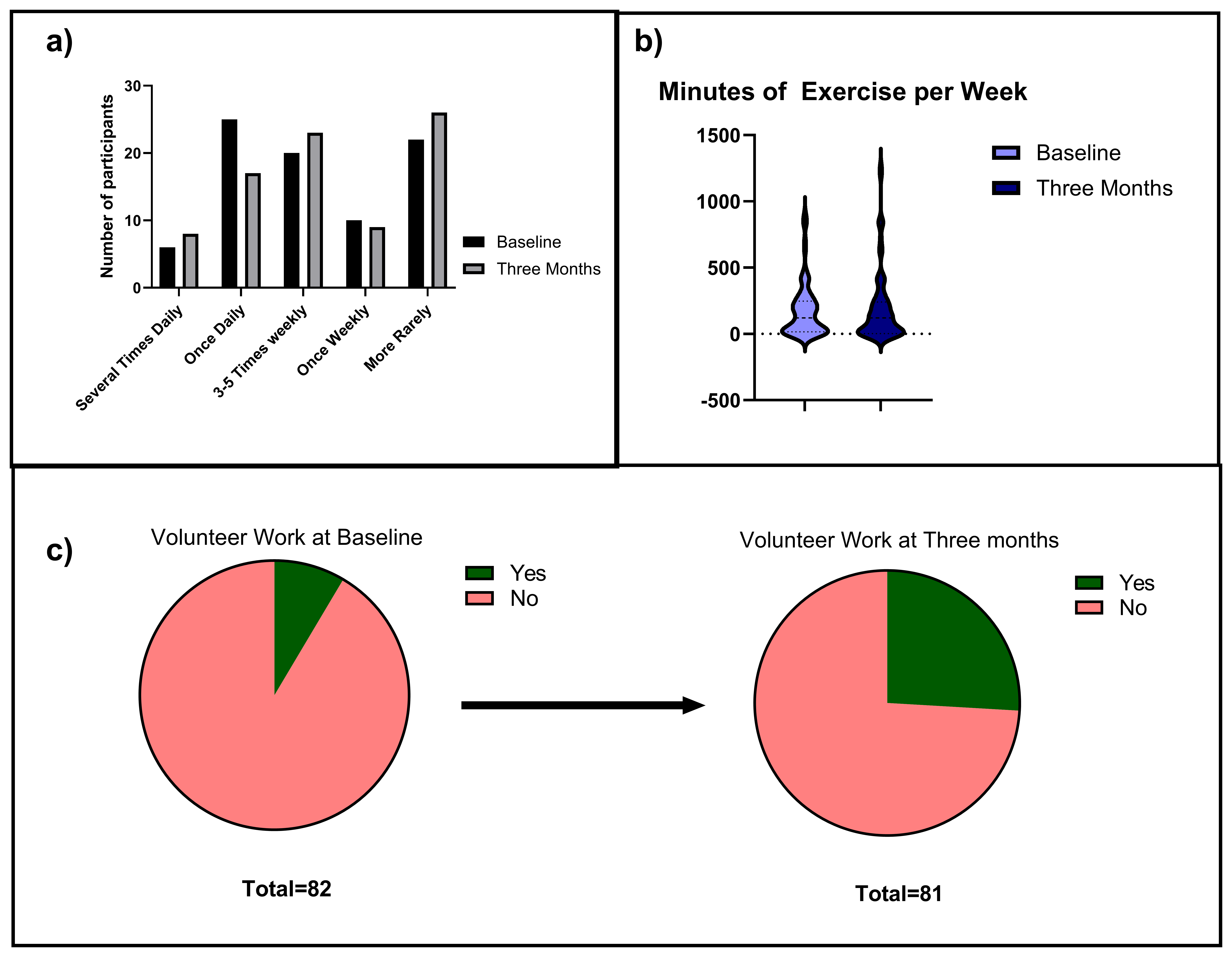

Supplement: Supplementary file 6 — Figure S2. Changes in work and exercise habits between baseline and 3 months. (A) Changes in frequency of exercise, with black bars indicating baseline and gray bars indicating 3 months. (B) A violin plot of changes in minutes of exercise per week. (C) A pie chart of the portion of the cohort that engages in volunteer work at baseline and 3 months, respectively. [file MDC3-12-922-s002.tif]
